# Progressive Strategy for Congenitally Missing Anteriors in Binder Syndrome: A Case Report

**DOI:** 10.7759/cureus.52426

**Published:** 2024-01-17

**Authors:** Lovely Bharti, Sunita Shrivastav, Ranjit H Kamble, Aksha Bhargava

**Affiliations:** 1 Orthodontics and Dentofacial Orthopedics, Sharad Pawar Dental College and Hospital, Datta Meghe Institute of Higher Education and Research, Wardha, IND; 2 Oral and Maxillofacial Surgery, Mahatma Gandhi Dental College and Hospital, Mahatma Gandhi University of Medical Sciences and Technology, Jaipur, IND

**Keywords:** cleft lip and palate, emotional trauma, maxillonasal dysplasia, psychological trauma, riding pontic, binder syndrome

## Abstract

Binder syndrome (maxillonasal dysplasia) is an uncommon congenital craniofacial condition. It is marked by distinctive facial characteristics including a flat, vertically oriented nose, maxillary underdevelopment, malocclusion, and nasal bone irregularities. This case study introduces an inventive strategy for addressing congenitally absent anterior teeth in a patient diagnosed with Binder syndrome. Our treatment approach combined orthodontic interventions and prosthetic restorations to enhance both aesthetics and function. This report explores the diagnostic and therapeutic aspects of the case, underscoring the encountered challenges and the ultimately successful outcome. This approach provides valuable insights into managing dental anomalies linked to Binder syndrome, emphasizing the necessity of a multidisciplinary approach for comprehensive patient care. A suitable strategy for adult patients might be slow maxillary expansion. This case report is about a rare case of maxillonasal dysplasia managed with the esthetic replacement of anterior teeth.

## Introduction

Binder syndrome was first described by Binder KH in 1962 as maxillonasal dysplasia [1]. It is a congenital malformation characterized by nasomaxillary dysplasia due to an underdeveloped midfacial skeleton. The etiology and pathogenesis of this syndrome remain unknown. Causes have included trauma or infectious factors, a family history, and neural crest cell abnormalities. This syndrome is distinguished by a flat nose, a convex upper lip with a short philtrum, half-moon-shaped nostrils due to a short columella, and an acute nasolabial angle [1]. Protruding upper incisors, an anterior open bite, crowding, and infrequently missing incisors or molars are common dental problems [1,2]. The disease affects 1 in 10,000 infants, impacting boys and girls equally, but it is probably under-identified, especially in certain ethnic groups. Binder syndrome is one of the rare disorders that clinically manifest with a midline cleft, along with the likelihood of missing anterior teeth due to the absence of the premaxilla, which requires critical orthodontic treatment planning and intervention (Venkatesh A et al., 2016) [1,2]. Patients with Binder syndrome also experience psychosocial trauma due to poor facial aesthetics caused by missing anterior teeth [3]. Depending on the patient's age, these cases may have already undergone several surgical operations, including bone grafting, maxillary advancement, and lip repair, which can further impact their psychosocial well-being [4]. The additional burden of missing anterior teeth, resulting in poor facial aesthetics, exacerbates their psychosocial burden. A significant portion of a patient's life spent with poor facial aesthetics can deliver a devastating blow to their psychosocial well-being and sense of self [5]. In such cases, orthodontic therapy is aimed at tooth alignment while leaving space in the edentulous area for a future prosthesis [6]. Once the orthodontic therapy is completed, the dental prosthesis can be created and placed. The psychological stress can be alleviated by carefully developing orthodontic treatment plans that are compatible with prosthetic rehabilitation [7]. It is possible to address the unattractive feature of missing anterior teeth temporarily during orthodontic therapy by providing a temporary prosthesis that will not interfere with orthodontic treatment mechanics [8]. Several methods are proposed to address this issue. Utilizing riding pontics in the treatment of Binder syndrome cases during orthodontic treatment may help reduce psychological trauma.

## Case presentation

**Figure 1:**
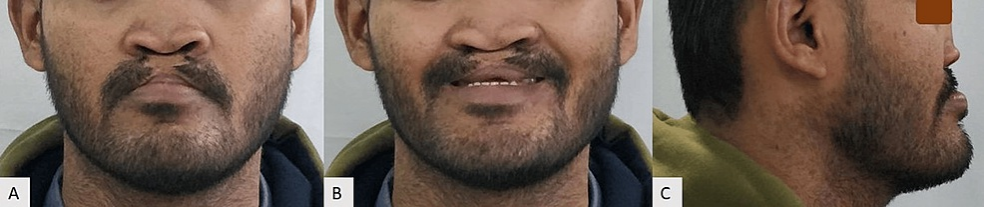
Pre-treatment extraoral photographs: (A) Frontal, (B) Smiling, (C) Profile.

A 25-year-old male patient reported to our department with the chief complaint of missing teeth in the upper front region of the jaw. Lip and palate repair surgeries were performed at the ages of 1.5 and 5 years, respectively. There were no significant maternal events prior to the patient's birth. During the extraoral examination, it was observed that the mid-face was hypoplastic, the nose was flattened, the profile was concave, and the nostrils were typically crescent in shape due to a short columella. The lips appeared competent at rest (Figure 1).

**Figure 2:**
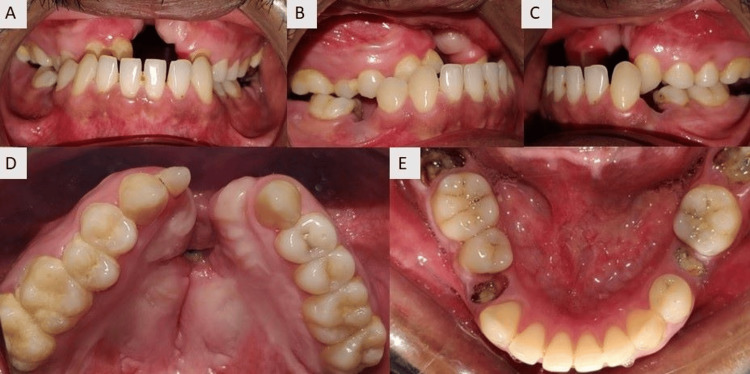
Pre-treatment intraoral photographs: (A) Maxillary arch, (B) Mandibular arch, (C) Anteriors in occlusion, (D) Left molars in occlusion, (E) Right molars in occlusion.

Intra-oral examination shows the absence of upper incisors (Figure 2A), palatal fistula on the left palatal region (Figure 2D), and multiple root stumps in the mandibular arch (Figure 2E).

**Figure 3:**
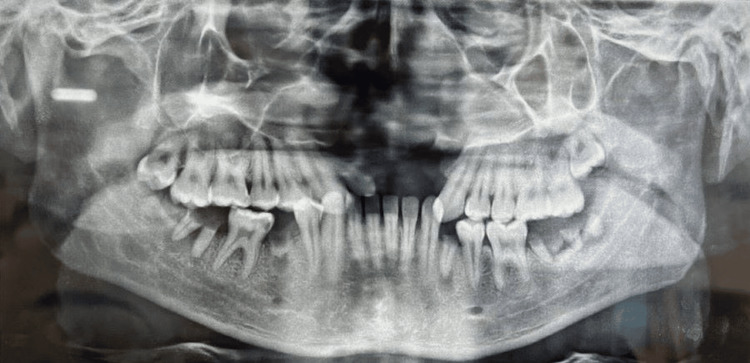
Orthopantomogram (OPG) showing missing 11, 12, 21, and 22; root stumps at 35, 37, 38, 46, and 48; supraerupted 17 and 27.

An orthopantomogram (OPG) shows the absence of maxillary anteriors and several root stumps in the mandibular arch; it also reveals supra-erupted maxillary left and right second molars (Figure 3).

**Figure 4:**
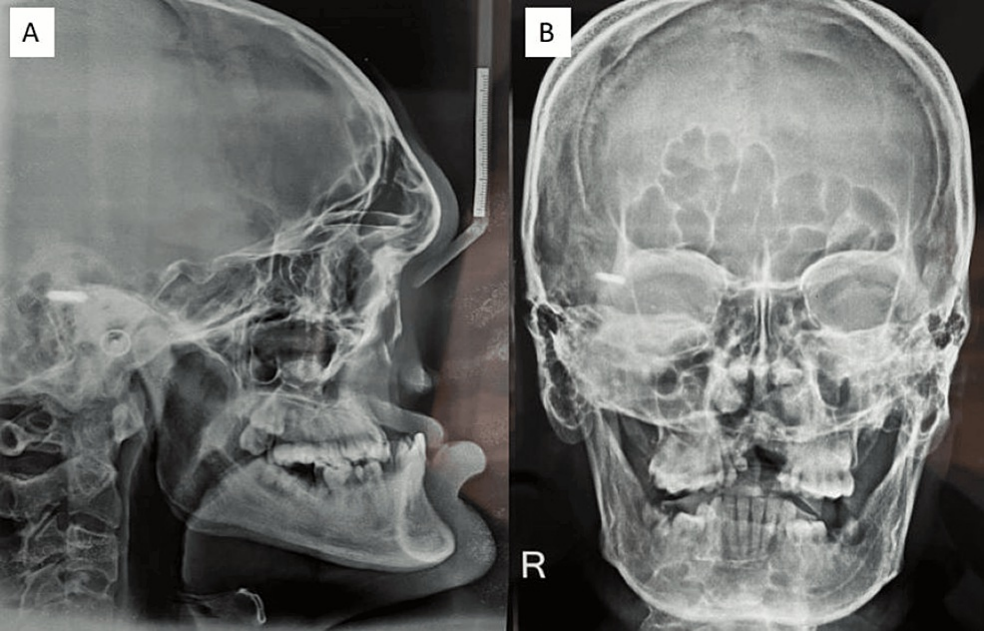
Pre-treatment radiographs: (A) Lateral cephalogram, (B) posteroanterior (PA) cephalogram.

The lateral cephalogram showed a Class III skeletal pattern, a concave profile, a retrognathic maxilla, a prognathic mandible, the absence of the nasal bone, and a prominent Anterior Nasal Spine (ANS) (Figure 4). The case appears to be indicative of Binder syndrome. Based on the clinical and radiologic examinations, the differential diagnosis included chondrodysplasia punctata, which is associated with scoliosis, not observed in this case. Another possibility was Robinow syndrome, but it is typically associated with short forearms and macrocephaly. After evaluation, the case was confirmed as Binder syndrome, which is consistently associated with the absence of the nasal bone, moon-shaped nostrils, cleft lip and palate, and dysplasia of the nose and maxilla.

Treatment objectives

The objectives of the treatment were the expansion of the anterior palate, constriction of the over-expanded posterior palatal segment, intrusion of the lower anteriors, extraction of root stumps in the mandibular arch followed by prosthesis placement, temporary restoration of missing anteriors in the maxillary arch, maxillary advancement, mandibular setback, and then rhinoplasty.

Treatment alternatives

The treatment alternatives for this case were the expansion of the anterior maxilla with a quad helix followed by the intrusion of supra-erupted teeth with fixed orthodontic treatment (0.022 MBT slot), decompensation of the maxillary and mandibular dentition for orthognathic surgery (maxillary advancement and mandibular setback), followed by rhinoplasty. Another alternative could be the expansion of the anterior maxilla with the help of a Nickel Titanium (NiTi) expander, followed by fixed orthodontic treatment using Roth prescription, distraction osteogenesis of the maxilla for advancement, and then rhinoplasty. In this case, a riding pontic-type prosthesis was made with an acrylic portion. As the edentulous space was not sufficient to place central incisor teeth, lateral incisors were used instead. Brackets were attached to the acrylic teeth. Simultaneous initial leveling and alignment, anterior expansion with a quad helix, and placement of the riding pontic anteriorly were done. To achieve progress, occlusion was dis-occluded with a blue bite on both first molars in the mandibular arch. This modality of placing the temporary pontic with an acrylic component not only improves aesthetics and restores patient confidence but also acts as a space maintainer for a future permanent prosthesis and prevents tongue thrusting.

Fabrication of the prosthesis

**Figure 5:**
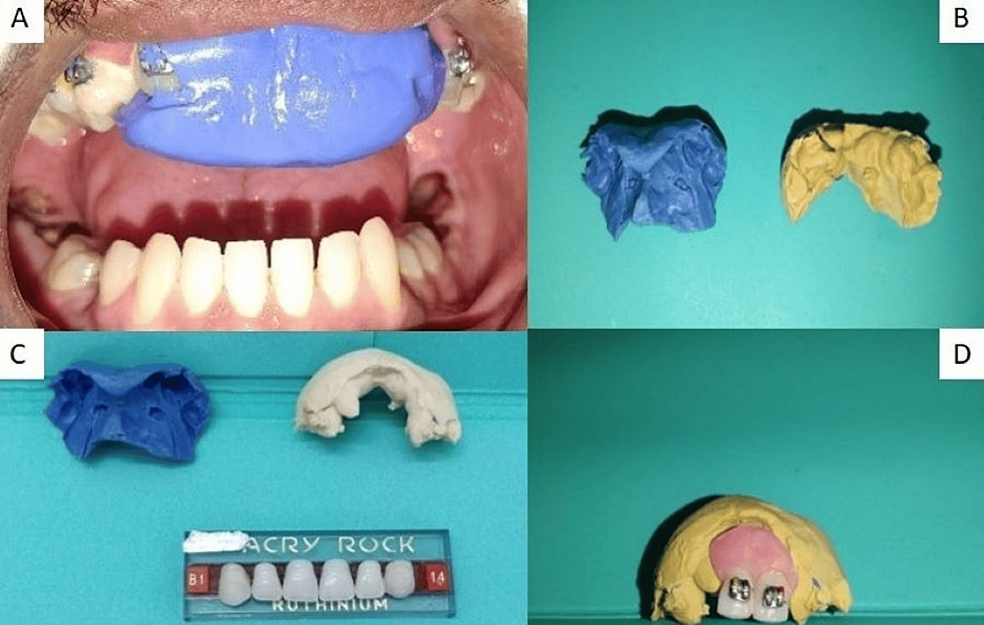
Steps of fabrication of prosthesis: (A) Impression using putty material, (B) Working model cast, (C) Selection of acrylic tooth, (D) Acrylic tooth secured with cold cure.

The process began by making impressions of both the maxillary and mandibular arches, followed by the preparation of models. A putty impression of the upper anterior region alone was taken (Figure 5A) and then poured with dental stone to create a working model (Figure 5B). Acrylic teeth, selected for their suitable shade and size, are shown in Figure 5C. A pink cold cure in the dough stage was applied, ensuring that the acrylic teeth were properly aligned with the arch (Figure 5D).

**Figure 6:**
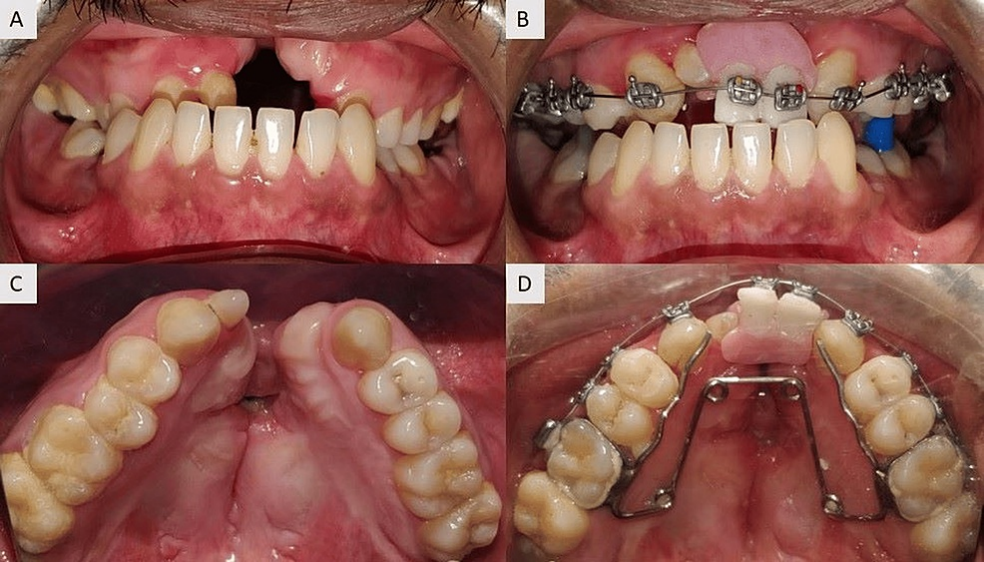
Pre- and post-treatment intraoral comparison: (A) Pre-treatment photograph of anterior teeth, (B) After placement of prosthesis in anterior region, (C) Pre-treatment maxillary arch, (D) After placement of quad helix for expansion.

**Figure 7:**
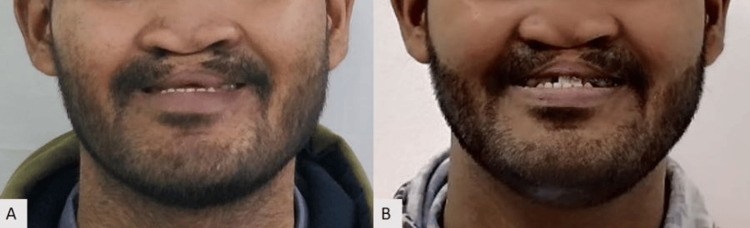
Comparison of pre- and post-prosthesis placement: (A) Pre-treatment extraoral photograph, (B) Post-prosthesis placement.

Brackets for teeth 21 and 22 were placed on the respective acrylic teeth by roughening the labial aspect and adhered using clear acrylic material. Both teeth were bonded together as a unit with the help of pink acrylic, which gives the appearance of gingiva. This assembly was transferred to the patient’s mouth and secured with the help of wire and modules (Figure 6). The pre- and post-prosthesis placement can be appreciated (Figure 7A and Figure 7B).

## Discussion

The maxillo-nasal dysplasia seen in Binder's syndrome is a generic abnormality of the maxillary and nasal areas, and it should be considered a distinct disorder or syndrome [5]. Although the disorder commonly occurs randomly without a specific reason, it may be associated with the disruption of the prosencephalic induction center during embryonic growth, resulting in such a disorder [6]. Furthermore, it has also been observed and proposed that disturbances in such induction centers may simultaneously occur in the vertebral regions, leading to a series of vertebral pathologies as comorbidities [7].

Facial features

Binder's syndrome primarily presents as an arhinoid face, characterized by a flat and vertically oriented nose due to the underdevelopment of the nasomaxillary structures [8]. Jawade et al. [8], in their study, mentioned distinctive facial features associated with this syndrome, including maxillary hypoplasia resulting in a reduced midface profile, severe malocclusion, a diminished nasal spine, atrophy of the nasal mucosa, and abnormal positioning of the nasal bones. While the absence of frontal sinuses can sometimes be present, it is not always the case [8]. Additionally, Binder's syndrome often presents with a concave facial profile, a reduced naso-frontal angle, and the possibility of hypertelorism [9]. The junction between the columella and upper lip is typically short and pulled back, lacking the usual triangular shape at its lower part. When viewed from below, the nostrils often exhibit a semi-lunar or crescent shape, and in cases of severe hypoplasia, they may appear triangular [9]. Skeletal underdevelopment is noticeable in the nasal floor, around the prenasal fossae, and the piriform aperture on both sides.

Skeletal and dental abnormalities

Skeletal changes associated with Binder’s syndrome have a direct impact on the patient's facial features. A high frequency of a missing anterior crest that separates the floor of the nasal cavity, along with the absence of a hypoplastic anterior nasal spine, is observed [9]. Consequently, the patient's facial profile appears flat with a lack of nasal prominence. Clinically, scaphoid depression can be observed, which is not visible in radiograph images, and the nasal bones are usually normal. Many studies have demonstrated hypoplastic or missing frontal sinuses, present in approximately 40-50% of cases with Binder's syndrome. Class III malocclusion arises due to the posterior positioning of the maxilla, accompanied by a short anterior cranial base. Additionally, there is an abnormal maxillary convexity [9,10]. Although not common, a few cases may be observed with a cleft palate associated with the syndrome. Delaire J et al. [10], in their study, reported the presence of microdontia of upper centrals and the absence of upper lateral incisors in patients diagnosed with Binder’s syndrome [10]. Patients undergoing orthodontic procedures are primarily concerned about their aesthetics, specifically their smile [11]. For an orthodontist, patient-related outcomes such as aesthetics and patient satisfaction are essential to build patient confidence and ensure compliance for a smooth treatment process [12,13]. In the present case, as the maxillary arch was observed to be collapsed, arch expansion was performed to address the issue. Consequently, an increase in spacing was clinically observed due to missing anterior teeth, which is one of the features of Binder’s syndrome. Providing a temporary prosthesis can address this problem, and it was implemented. Subsequently, orthognathic surgery will be planned for the patient following adequate orthodontic corrections [14]. Motivating patients during treatment is crucial, as it can lead to good adherence to the treatment plan, resulting in improved clinical outcomes. A significant clinical challenge when using long-span arch wires is mucosal irritation, speech difficulty, and unsatisfactory aesthetics [15]. Currently, the patient is regularly scheduled for follow-up visits to ensure that the proper and desired steps are being carried out as planned for maxillary advancement surgery, followed by post-surgical orthodontics. Performing the procedure on the dental chair has minimized psychological stress on the patient and saved significant treatment time. Additionally, the required materials are easily accessible, and the fabrication steps are less cumbersome. These aspects contribute to addressing the problems more efficiently.

Economical background

Binder syndrome, though relatively rare, presents unique economic considerations in the realm of healthcare. The management of this congenital craniofacial anomaly often involves a multidisciplinary approach, including orthodontic, surgical, and prosthetic interventions. These treatments can lead to substantial healthcare costs, including consultations, diagnostic imaging, surgical procedures, orthodontic appliances, and prosthetic devices [16]. Additionally, Binder syndrome may result in lifelong healthcare needs as patients may require ongoing dental and orthodontic care, as well as periodic revisions of prosthetic restorations to accommodate growth and changing oral health needs. These recurrent expenses can place a long-term financial burden on affected individuals and their families [17]. The economic impact extends beyond the patient level to include healthcare systems and insurance providers. Healthcare institutions must allocate resources for specialized clinics and teams capable of diagnosing and managing Binder syndrome effectively. Insurers may face challenges in covering the wide range of treatments required, especially given the rarity and complexity of the condition.

## Conclusions

In conclusion, this case report highlights the distinctive features and challenges associated with Binder syndrome. The treatment approach involved a collaborative effort among various specialties, such as plastic surgery, orthodontics, and speech therapy. The strategy adopted in this case demonstrates effective treatment and its outcomes in syndromic patients with missing teeth undergoing orthodontic treatment. Other clinical advantages of this procedure include reduced complexity and time-saving, which are crucial in orthodontic treatments. Additionally, it enhances patients' confidence in the treatment outcomes and improves patient compliance.
